# Book Reviews

**Published:** 1821-10

**Authors:** 


### CRITICAL ANALYSES

OF

### RECENT PUBLICATIONS, IN THE DIFFERENT BRANCHES OF MEDICINE AND SURGERY.

"I would have men know, that, though I reprehend the easie passing over of the causes of things " by ascribing them to secret and hidden vertues and properties; (for this hath arrested and laid " asleepe all true enquiry and indications;) yet I doe not understand but that, in the practical " part of knowledge, much will be left to experience and probation, whereunto indication cannot " so fully reach: and this not only in *specie*, but in *individuo*. Yet it was well said, *Vere scire* " *esse per causas scire*."—BACON.

*Elements of Medical Logick, illustrated by Practical Proofs and Examples. The Second Edition, with large Additions, particularly in the Practical Part.* By Sir GILBERT BLANE, Bart. Fellow of the Royal Societies of London, Edinburgh, and Göttingen; Member of the Imperial Academy of Sciences of St. Petersburgh; and Physician to the King. 8vo. pp. 265. T. and G. Underwood, London. 1821.

A RECENT French critic, speaking of a new edition of SCARPA's work on Diseases of the Eye, very properly observes of it—" Que ce n'est point de ces editions que la supercherie de nos libraires fabrique en recomposant la feuille du titre." Precisely the same remark may be made respecting the new edition of the work before us; for, it is not only enlarged by many useful, and in some points necessary, additions, but is judiciously modified, as we are convinced, throughout the greater part of its former arrangement. The author himself, indeed, in the advertisement prefixed to the new edition, states, that he felt bound, in gratitude and duty, from the success of the first impression, in 1819, to render the present one still more worthy of public acceptance, by large additions.

Logic, as one of the moral sciences, has been so much perverted by the schoolmen, that the very name of it excites a smile. BACON was so convinced of this, that he studiously avoided the very name of *logick*, and either substituted for it the synonym *dialectic*, or employed his own quaint peryphrasis " novum organum."

In undertaking the present work, our author has attempted to restore logic to its original dignity and utility; and, when we consider how important it is to those who are to practise physic, that they should possess the means of disciplining their minds to the right investigation of truth,—the profession will feel obliged to Sir Gilbert for having endeavoured to extend the art of discriminating good from bad evidence, to a branch of natural knowledge essentially beset with every species of fallacy.

That such is the nature of the obligation we owe to Sir Gilbert Blane, may indeed be collected from his own declared purpose, with which the introduction terminates:

"It is the author's intention, with unfeigned diffidence and humility, to endeavour to point out in what medical truth and error consist; what are the difficulties that have obstructed the progress of the art, and what are the means of obviating them: in other words, (if he may be allowed to adopt professional technology,) to expound the physiology, pathology, and therapeutics of the medical mind, as the result of fifty years' observation, experience, and meditation, on these subjects."

In the preliminary remarks, the author has entered into a concise metaphysical inquiry into the faculties of the mind employed in ascertaining those truths which depend on correct observation and a right interpretation of the works of nature. He asserts, in common with some other authors, that the senses may be considered as the types of universal nature; and he further remarks, that there is a like accordance with the faculties of the mind, particularly that part of the mental constitution by which we are made susceptible of habit and association.

"In tracing this still farther, we perceive that, by virtue of this correspondence or co-ordinance of the frame of the mind with the established course of nature, there is, in all the changes produced by the action of external bodies on each other and on our own bodies, a rapid and instinctive connexion between cause and effect, manifested in that part of our constitution by which it is made susceptible of habit and association, and which is indispensable to our well-being, and even our existence, particularly in early life. This may literally, and without a figure of rhetoric, be termed the mental organ; for it carries a reference to the constancy of nature, just as the eye does to the affections of light, and the ear to those of the air. Thus is every organ and function of the body, and every faculty of the mind, co-relative with, or represents and reflects, as it were, not only the elements, but the laws, of universal nature;* so that the sublime images and glories of the creation are displayed to our sensitive capacities as objects of grandeur and beauty, and to our intellectual capacities and enraptured minds as irresistible evidences of harmony and design."—P. 20—21.

In the body of the work, the author sets out with professing to follow the inductive method of investigation, as less liable to error than the syllogistic. This last, however, he does not treat with the same contempt as some other modern metaphysicians; alleging, that it tends "to give precision to language and thought, and to induce habits of close attention and patient ap-

* See this sentiment more fully illustrated in a Lecture on Muscular Motion, page 40, read before the Royal Society, 1788, by GILBERT BLANE, M.D. It is also ingeniously and appositely alluded to in Mad. DE STAEL's account of the German poetry, in the work entitled *De l'Allemagne*, 1815.

plication of mind;" at the same time lamenting that it should have superseded all other learning, for more than one thousand years, in the schools of Europe.

He next enters into a comparison of the different degrees of certainty and of difficulty in the ascertaining of truth in physical researches. Of these, Sir Gilbert thinks that chemistry is the most exact, so that a single experiment may ascertain a general truth, there being only one *affecting* cause, namely affinity: whereas, in mechanical philosophy, and still more in all that relates to organic nature, there is a combination of many causes, which more or less tend to modify the results. With a view to such researches, therefore, he alleges, that all these causes, whether concurring or counteracting, should be specified. This, the author contends, has never been done; and he therefore attempts it, by enumerating the principles which belong exclusively to animal life, and which he not unaptly designates as the *alphabet*, or elementary constituents, of physiology and pathology. These elementary principles are ten in number:—1, The Generative; 2, the Conservative; 3, the Temperative; 4, the Assimilative; 5, the Formative; 6, the Restorative; 7, the Motive; 8, the Sensitive; 9, the Appetitive; 10, the Sympathetic."

To follow our author through his detailed illustrations of each of the above principles, would be, in a great measure, to travel over the same ground we did on a former occasion, when we reviewed the first edition. We cannot, however, deny the public and ourselves the pleasure of noticing a few of the many facts and observations, some new, and some more happily demonstrated than heretofore, with which the body of the present edition abounds, and which we find arranged under the different subdivisions that treat of the elementary principles. Thus, under the "generative," there are some curious remarks of the steady proportion of the sexes, in spite of the great inequalities of individual procreation; and, in speaking of the "conservative" principle, Sir Gilbert remarks, that, though one of the fundamental principles of life, it has been strangely neglected, and even entirely overlooked, by some physiologists. As for the attempt made by several of them to account for this curious principle by the perpetual motion of the living fluids and solids, the author considers it as futile in the extreme. We have, under the "temperative" principle, considerable additions of importance, particularly where the author combats those opinions which assign a chemical origin to animal heat. On this subject, Sir Gilbert's remarks are worthy of attention.

"The standard of heat is very different in different species of animals. In the amphibia and fishes, it is very little above that of the surrounding medium. But the resistance which these animals give both to heat and cold, by maintaining their specific temperature in spite of the application of higher or lower degrees of it, contrary to the law of communication in inanimate bodies, is a proof that temperature is both raised and depressed by some power essentially inherent in life. This is most observable in birds; for, in those even of the smallest size, the natural heat is ten or twelve degrees above the human. When it is considered how immeasurably greater the abstracting power of the atmosphere is in these small bodies, in consequence of the ratio of their surface being as the square of their mass, it is utterly impossible to account for this on chemical principles, and must depend on a specific generating power, furnished in various degrees to the respective species of animals; and it must be astonishingly great in small animals, to enable them to resist the strong power of abstraction in the external medium. This argument is rendered still more strong by what is found to take place with regard to some insects. Let the bulb of a thermometer be thrust into a swarm of bees, the heat indicated will be 97° or 98°, that is, as high as that of the living human body.*—P. 43—44.

We also wish to recommend to our readers a number of other remarks on temperature, physiological, pathological, and practical, with which this part of the work abounds, and many of which are new.

It is under the appellation of "assimilative" principle that the author has grouped the two physiological functions of digestion and secretion. Here Sir Gilbert enters at full length into the changes produceable by the sole power of animal life, as distinguished from the chemistry of dead matter; alleging that, from the similarity of animal matter in all animals, whatever their food may be,—also from the experiments of Allen and Pepys on respiration,—the power of life in producing, or rather creating, new modifications of matter, is far more considerable than has been commonly allowed. Sir Gilbert takes occasion also to advert to the question respecting the influence of the nerves in modifying digestion and temperature, and seems unwilling to admit it in the sense and to the extent of some other physiologists; grounding his opinion, not only on what occurs in animals without brain and nerves and in vegetables, but on a distinction also which should be made between an *actuating* and an *influential*, or *intervening*, cause.

In a book of so much merit, and one which for reasoning power has seldom been surpassed, we are, of course, anxious that nothing should wear the appearance of wrong induction. An instance, however, of want of *rectilineal* induction, as it appears to us, occurs under the very head of which we have just given an analysis, and relates to the author's individual opinion regarding the influence of nervous action on the assimilating process. The author alleges, first, that "nervous power is found, in some instances, even to retard and disturb the assimilating process;" and he supports his assertion by the fact that, in many cases of hemiplegia, digestion goes on better than in ordinary health. Now, although we do not mean to deny that either of these premises may be correct, it does not follow that, *ergo*, where there is *no hemiplegia*,—or, in other words, where the " nervous power is not withdrawn or impaired,"—nervous action will be found "to retard and disturb the assimilating process;" a conclusion to which the reasoning of the author seems to us inevitably to lead, though, we believe, without the consciousness of the writer. Besides, Sir Gilbert is aware that, where a portion only of any symmetrical arrangement in animal organization has its function suspended, *par ure cause quelconque*, its power of acting is *generally* transferred to the agnate portion whose vitality has remained entire. That which would be a much better illustration of Sir Gilbert's proposition, (were the fact true,) and which Sir Gilbert has actually advanced as " a further illustration," on the authority of an article in the 18th Number of the Journal of the Institution, is the continuation of digestion and chylification after the division of the nerves of the stomach. Unfortunately, such is not really the fact; for, from the repetition of the same experiments which has taken place at the Royal Institution, subsequently to the publication of Sir Gilbert Blane's new edition, we have been taught to believe that digestion and chylification do *not go on* when the nerves of the stomach are properly divided: that is to say, when a portion is removed from the length of the said nerves, so as to preclude the chances of apposition in the divided ends. Does not the individual who has had the misfortune of injuring the sacral nerves of both sides, by falling backwards on the sharp edge of a step, lose the power of moving both extremities? and has it not been remarked, by various writers, that, where the one extremity only has been paralyzed, the other has exhibited signs of evident increase in its temperative, assimilative, motive, and sensitive principles?

There is another doctrine of the author in this part of the work, to which we are not disposed to subscribe. We are the more anxious to mention this, because it relates to practical medicine, the improvement of which must, after all, be the principal object of the author, of ourselves, and the public. Sir Gilbert observes, at page 67, that, " as fluids are incapable of assuming an organized structure while they retain that form, and therefore incapable either of initiating or giving direction to motion,—all the initial actions of life, as well as its ulterior processes, must be referred to the solids."

Now, admitting this proposition to be correct, how does it follow " that the virtues of medicine should be directed to those attributes that belong to solids; that is, excitability, sensibility, and contractibility?" We confess we are not ourselves so entirely divested of early prejudices in favour of humoral pathology, as not to see that the *solidism* of the present day has done nearly as much harm in physic as the former doctrine. Sir Gilbert himself has acknowledged that certain medicines operate on principles purely chemical; and as, in the two examples of this fact adduced by him, *fluids* alone are concerned in producing the very diseases which are only removed by chemically correcting, neutralizing, or annihilating those fluids, it follows that "sensibility, excitability, and contractibility," are not the only principles to which the virtues of medicines should be directed.

Some people have contended that the "formative," or plastic, principle does not differ from the "assimilative;" but Sir Gilbert compares the one to an edifice, and, to the materials of which that edifice is composed, the other. The creation and application of these materials to the rearing of the wonderful fabric of the living body, is one of the most astonishing phenomena which the human mind can contemplate. There are some practical remarks, with which this article concludes, respecting the expediency of bleeding in old age,—a period of life in which the author states that less blood is expended in the repair of the body, and is therefore liable to accumulate and produce plethora, requiring evacuation. It is from the cause above mentioned that aged people are frequently subject to spontaneous hemorrhages, which are not only innoxious but salutary.

"I was lately called to a lady aged 82, emaciated and weak, labouring under a profuse hemorrhage from the nose, by which nearly a quart of blood was lost. It was followed neither by faintness nor weakness, but by an improvement in health, in point of vigour and alacrity, evidently proving that there was a redundancy of blood, the removal of which gave relief. Other similar cases have not unfrequently occurred to me. Lately I had occasion to know of a female, aged 100, who, in an attack of pneumonia, had been freely and successfully bled in the arm. Sydenham gives very strict cautions against bleeding aged people, without assigning any reasons, and without any exceptions or qualifications; resting, no doubt, on the plausible notion that, old age being a state of exhaustion and debility, a loss of blood must always be detrimental. This is perhaps true in a majority of cases; but I am well convinced that practitioners will fall into frequent and fatal errors by adhering to it as an invariable rule."

We read with pleasure the new remarks on sleep, contained under the head "restorative principle." They are illustrated by practical observations and anecdotes; nor were we less entertained with what he has written on the subject of the *vix medicatrix naturæ*, which is treated at some length.

In discussing the next principle, the "motive," or muscular action, in its most extensive sense, Sir Gilbert has introduced some physiological, as well as practical, remarks on tension, as a state of the living fibres absolutely necessary for their healthy action. The detrimental, and even fatal, effects of its defect are exemplified in hemorrhage and sudden depletion; while the salutary results of even artificial tension are illustrated by swathing the body and the strapping of ulcers. It is upon the principle of artificial tension, we have a right to presume, that our author has imagined a mode of treating chronic and threatening hydrocephalus by bandages; the good results of which our readers will find illustrated by examples, in the paper addressed to us by the author, and inserted among the Original Communications. Its further advantages (tension) in promoting digestion, are thus developed by the author:

"Nature has wisely provided that, along with the pure nutritious matter of the food, there should be a certain admixture of unassimilable matter, in order to give it more bulk, and thereby more tonic energy to the stomach. The most invigorating articles of food, accordingly, are such as are introduced into the stomach in a solid form, and not only devoid of fluidity, but possessing a certain degree of hardness and tenacity, so as to excite the powers of the containing viscus to stronger action. It is found, therefore, in the human species, that plain solid food, combined with a certain proportion of unassimilable matter, is infinitely more efficient for the purposes of health and strength, than that which consists of pure alimentary matter, whether gelatinous, albuminous, oily, or saccharine. And, with regard to animals, it is a well ascertained fact, in horses, that their strength is much better sustained by hay than by grass; for the stomach, being an organ of universal sympathy, does, by the exertions on which it is put in digesting hard food, confer vigour on the whole frame."

A very important obstetrical question, and new in this edition, will be found treated under this head at full length. We allude to the occurrence of uterine hemorrhage, or flooding, particularly after child-birth. On this subject, Sir Gilbert lays a great stress on the necessity of administering cordials and opium; and he alludes to five cases which occurred in his own practice, in four of which he made use of that plan of treatment, with the most satisfactory results. With the usual caution which invariably marks the man of long-tried experience, in advancing these cases, Sir Gilbert does not mean to generalize their mode of treatment: and in this he is perfectly right; for we can assure him, that in our own not very limited practice, in cases of uterine hemorrhage, an indiscriminate use of cordials, or even of opium, would have frequently given rise to the most fatal consequences. As to the reliance of the author upon cordials in assisting to expel the placenta, no practical obstetrician, we trust, will ever be found to entertain such a doctrine. Where there is any hemorrhage to an alarming amount before the placenta is expelled, it is the duty of the accoucheur to remove it forthwith by manual exertion. Time here becomes an idle question; for this operation may with safety be performed the very next minute after the delivery of the child, without waiting, according to the mistaken practice of Hunter, for the unassisted efforts of nature. But the removal of the placenta alone does not, in many instances, put a stop to the hemorrhage; for the uterine contractions, upon which the stopping of such hemorrhages depend, are occasionally too feeble or too tardy. In such cases, ere the accoucheur has recourse to cordials as his only auxiliary, he should stimulate the womb to action, either by irritating it with his finger gently introduced, or by grasping it from without and through the relaxed parietes of the abdomen. It is incredible in how many cases of flooding, under similar circumstances, this last simple operation has proved successful. It is in cases of flooding from miscarriage at an early period of pregnancy, when the hand cannot be introduced to bring away the ovum, and the hemorrhage has been so great, and has lasted so long, as to endanger the mother's life, that cordials and opium combined will be found of immediate necessity and great assistance. We recollect, some years ago, saving the life of a young married lady, to whose assistance we had been called by Dr. Paris, and whom we found almost expiring from the enormous quantity of blood she had lost during the process of miscarriage, at a very early period of pregnancy. In this case we kept administering, with our own hands, (for it was a case admitting of no dependance upon the zeal of mere attendants,) large draughts of brandy, as well as wine, with laudanum, every hour, during the whole of a long winter's night. In another instance, which we select from many more, because of a more recent date, a married lady, some miles from town, was seized with uterine hemorrhage, when about six or seven weeks gone with child, as she supposed. She had been blooded before our arrival, and the hemorrhage had increased to a most alarming extent. We prescribed port-wine, of which the patient took three bottles in the course of the morning: the flooding was thus arrested, and in three or four days afterwards she expelled a false conception, of the size of a very large hen's egg, extremely vascular. It is curious to remark with what facility women in such cases recover their strength after losing so much blood, when they have been treated by means of cordials, opiates, and wine.

Our readers will readily subscribe to the truth of Sir Gilbert's ingenious remarks on the necessity of exercise to the growth, strength, and health of the various organs, with the considera-tion of which he concludes the article on the motive principle. We must be indulged in the pleasure of quoting a short extract from our author, on the subject of indolent habits and excess of sleep.

"The indulgence in indolent habits and excess of sleep in which those ranks of life who do not depend on bodily labour are enabled to indulge, contribute, no doubt, to create those diseases, particularly the gout, which are peculiarly incident to the affluent. During the twelve years in which I was physician to one of the largest hospitals in London, not one case of gout occurred in several thousands which came under my care; an irrefragable proof that, as labour (I ought to say, habitual and unremitting labour), with simple diet, infallibly prevents gout, so its remote cause must be sought for in the reverse of these. Of the manner in which this is brought about,—that is, the proximate cause, I have never seen any rational conjecture, nor am I able to form one myself; but such is the fact. The share which excess of sleep has in creating chronic disease, has probably not been sufficiently attended to, unless we are to except the common popular observation, that all long livers have been early risers. Ought not a superfluous share of sleep to be deemed a debauchery, as much as an excess of food and drink? If the proximate cause of gout and hypo-chondria should ever be discovered, it will probably be found in the relation in which defect of muscular action and excess of sleep, com-bined with a redundancy of aliment and the habitual stimulation of vinous liquors, stand with regard to the symptoms which characterize these maladies."

There is nothing very new in the present edition regarding the "sensitive" principle; but we have another principle added to the former list, under the appellation of "appeti-tive." Some curious examples are adduced under this head, of the existence of this principle in vegetables,—such as their instinctive motions in search of light, air, heat, and water.

The last article in the list of vital principles is "sympathy," which, by establishing the mutual influence of every part upon every part, constitutes the unity or individuality of animals. The author alleges, that, though sympathy be carried on, in general, through the instrumentality of the nerves, it is not ab-solutely nor invariably so; and this he has illustrated by facts adduced from the economy of vegetable as well as animal life.

Having thus established an analytical scheme of the princi-ples of healthy life—an alphabet of physiology,—the author thinks he has laid the foundation for the genuine data of theo-retical medicine; since, as he observes, the elements of disease can be expounded only by a thorough knowledge of the ele-ments of life and health. He, however, does not pretend to have effected this with any thing like perfection; but relies on future investigators for the completion of a system which cannot fail to add dignity to our professional avocations.

The proper object of the present work, however, is introduced in a more particular manner with the second section, wherein the various causes that have most materially obstructed the improvement of medicine are enumerated and elucidated. The order in which these causes, or sources of error, as the author calls them, are considered, is as follows:

"1. The fallacy and danger of hypothetical or theoretical reasoning.

"2. The diversity of constitutions.

"3. The difficulty of appreciating the efforts of nature, and of discriminating them from the operations of art.

"4. Superstition.

"5. The ambiguity of language.

"6. The fallacy of testimony; with some remarks on the excessive deference to authority."

These important subjects are treated in as many sections, with a variety of reasonings and practical illustrations. The great leading principle which pervades the whole, and which it appears here that the author is very anxious to inculcate, is, that the various phenomena of vital existence, whether animal or vegetable, rest on laws of nature quite distinct from those of inanimate matter. The false theories, and the nugatory or pernicious practice built upon them, in the seventeenth or beginning of the eighteenth century, were occasioned by a misapplication of the mechanical and chemical philosophy, and by overlooking the energies peculiar to life.

"After the revival of genuine philosophy in the seventeenth century, it might naturally be expected that medical science would immediately avail itself of its light, and partake of its benefit; but this was so far from being the case, that, in the first instance, it proved a new source of error, and threw fresh impediments in the road which was supposed to be thrown open to the improvement of rational medicine. The discovery of the circulation of the blood may, indeed, be considered as one of the first fruits of the inquiries into nature begun in that age. But, though this is a fundamental element in the economy of the living body, it throws little or no light on the principles peculiar to life, being purely of a mechanical nature; and, abstractedly considered, hardly admits of any application to the practice of medicine. On the contrary, this discovery, by its perverted application, tended to corrupt and mislead, by a loose adoption of the principles of mechanical philosophy, so well laid down in that age by Galileo and others. Borelli, in investigating the force of the heart by experiment, estimated it at 180,000 pounds; Hales, at 51 pounds; Keil, at 1 pound. The mechanical powers of the stomach were, about the same time, subjected to experimental research by Pitcairn, who gravely gave out that he found this viscus, in the human subject, exerted a force equal to 12,900 pounds, in compressing food in the process of digestion. Others, conceiving that chemical power had the chief share in this function, endeavoured to evince that the change in the food was brought about by means of heat and fermentation. Sounder principles have referred these changes to powers which have nothing in common with the mechanical and chemical powers which characterize inanimate nature."

The influence of Boerhaave, and the singular predominance which his theories maintained all over Europe for half a century, are adverted to by our author, who exposes the equally indefensible theories of Hippocrates, Galen, and Sydenham, as affording melancholy evidence of the aberration of the most powerful minds under particular circumstances.

Having read thus far, we began to fear lest our author should next endeavour to prove the futility of our anatomical and physiological studies, and the superiority of empiricism over them: but we find him, very agreeably, turning round upon us as the advocate of rational and philosophical medicine, in seven very powerful arguments in favour of the latter, so as ultimately to bring the dogmatics and empirics into a friendly compromise.

In a work of this nature, we would naturally expect a strong exhortation on the necessity of experience and practice, as the most indispensable of all acquirements; and we are not disappointed.

"It is by practice we learn to connect cause and effect, means and end, operations which, in well-turned minds, are performed with promptitude and precision, by interpreting fairly the appearances of nature, and stripping them of those adventitious fallacies which mislead ordinary minds. In order to attain this, there are required an appropriate natural capacity, the good fortune of not having been beset with prejudices in early life, an habitual exercise in the observation of nature, a candid and ingenuous disposition, an ardent love of truth, an exalted sense of duty, a large store of facts in a correct and tenacious memory,—the power of combining, comparing, and discriminating these, by an intuitive glance, in the moment of applying them to the practical end in view. This is what is understood by the term *tact*, in English and French, εὐστοχία, in Greek; being that faculty by which practical facts are decided on, and is performed by an instantaneous, silent, and almost unconscious calculation and induction, to be met with only in minds at once happily constituted and highly cultivated."

And again, in continuation of the same subject:

"From this it will be seen how vain all acquired knowledge is, without practical habits; for, in the liberal as well as in the mechanical arts, expertness can be obtained only by frequent and long-continued exercise of actual labour; and it is by a happy and appropriate figure that those who become skilled in languages, painting, eloquence, physic, or the common business of life, are said in Latin, *callere*, whence *callidus*; words derived from *callus*, that is, a horny substance formed on the hands of mechanical artisans, by long and unremitting labour. Whatever the attainment may be which we aim at, whether mental or manual, nothing but practice will make perfect; there being a certain expertness in the exercises of the mind, as there is a slight of hand in mechanical operations, attainable only by long and assiduous application. This same law of nature is finely illustrated in the following passage from CICERO *de Officiis*:—*Nec* MEDICI, *nec imperatores, nec oratores, quamvis artis præcepta perceperint, quidquam magnæ laudis dignum, sine usu et exercitatione consequi possunt.*"

Section the fourth, on the "diversity of constitution," is full of practical matter. The main foundation of this diversity is the artificial circumstance of constitution and habits of life induced on the human subject by the exercise of reason. The principal aim of this part of the work is to throw out cautions against indiscriminate practice. One of his illustrations is taken from Dr. Hamilton's work on Purgatives, which is here praised, but also considerably censured on this score; Sir Gilbert taking occasion, at the same time, to differ from that popular writer on the subject of specific or elective purgatives. Our limits, however, do not admit of our dilating on this and other practical points discussed with equal felicity under this head.

On the third source of error, the author is considerably more diffuse than in the former edition; and, in discussing the question on the comparative curability of diseases, we find the following new passage:

"The author submits the following outline as the basis of a more general rule on this subject. It is founded on a classification of diseases as they affect the three great vital cavities of the body. Those of the head, such as epilepsy, mania, hemiplegia, and hydrocephalus, seem to be the least under the control of art, owing, probably, to the very delicate texture of the brain: those of the abdomen, on the other hand, such as inflammation of the bowels, bloody flux, and cholera, afford us proud triumphs of medical efficiency; for it will be conceded by the most sceptical, that, without the intervention of art, a great majority of such cases would prove fatal: those of the thorax, intermediate to the other two in situation, are also intermediate as to the degree in which they are medicable, the chief of them being inflammation of the lungs, asthma, and consumption; the two first affording the most unambiguous proofs of life being frequently saved by a vigorous interposition of medical agents, while the last bids defiance to all the resources of art."

In exhorting the reader to acquire, if possible, some definite ideas regarding the limits between nature and art, Sir Gilbert concludes with the following terse remark:

"For, if his mind had a bias to scepticism, he might on some occasions be unable to satisfy himself, in case of a fortunate result, whether his patient had recovered by *virtue* of the means employed, or in *spite* of them; and, in case of a fatal result, his feelings would be still more distressing; for what could be more painful to a conscientious and sensitive mind, than the uncertainty whether the loss of the patient was most imputable to the remedy or to the disease. If, on the other hand, he should be prone to credulity, he might be so far blinded as, *bona fide*, to plume himself, and to congratulate his patient on a great *cure*, in what may have only been a great *escape*."

The section on "superstition" is full of classical and historical allusions, and will, perhaps, prove the most interesting to those who read for mere amusement; though this amusement must be considerably abated by the humiliating picture of the imbecility of the human mind, as exemplified in the history of fatalism, witchcraft, incantations, and quackery.

The article on "ambiguity of language," as a source of error in medicine, is very instructive. Of the many exemplifications of this error brought forward by Sir Gilbert, we shall only select that of "scurvy," a term applied to certain cutaneous affections, and also to the "sea-scurvy," a disease having nothing in common with the other. Boerhaave himself confounded them so effectually as to cause four hundred patients labouring under sea-scurvy to be treated with mercurial frictions; all of whom perished.

In adverting to the controversies respecting the yellow-fever, the author alleges that they have arisen chiefly from an ambiguity of language; on the grounds that, although the diagnostic characters of this disease are to be found in that form of it which arises from endemic exhalations and in the sporadic form, there is occasionally, superadded to these, a typhoid cause, which renders it contagious. The whole article on yellow-fever is considerably curtailed in the present edition, without losing any of that importance which the pen of our author knows how to impart to subjects of such moment.

The last source of error enumerated is the "fallacy of testimony." The reader need not be told how deceitful is the mind of man, which does not even know its own motives, and, from education, early prejudices, or self-interest, embraces the most glaring absurdities and falsehoods as truth. It is, therefore, entirely in the spirit of such a work as the one before us to advert to the errors which have arisen from the intentional, but still more from the *un*intentional, mis-statements of matters of fact by writers. Sir Gilbert's illustrations on this head are also taken from practical subjects, among which is introduced the history of colchicum as a cure of gout, and a reflection on the advantage of scientific knowledge, if correctly recorded.

We have omitted, in our account of this useful work, any notice of the many moral and philosophical allusions and illustrations, as they relate to general science; but shall give the reader a specimen of them, by transcribing the two last pages of the author's conclusion.

"From the picture that has been exhibited of the innumerable doubts and difficulties which clog the attainment of medical knowledge, and embarrass the application of it to practical purposes, the timid, sceptical, and indolent, may be discouraged from studies apparently so arduous in their prosecution, and so questionable as to the efficiency and utility of their result. But it is not from characters of this description that any good can be expected in any of the useful arts of life. If a like despondency were to pervade mankind in general, there would be an end to all that enterprize and energy which alone can enable them to act up to their destiny, and follow up those pursuits upon which the perfection of their nature depends. As the senses would have lain dormant for ever had there been no external objects to stimulate them, so the faculties and virtue which characterize rational nature and civilized life could never have been developed, but through the excitement of those pains, wants, difficulties, and dangers, inseparable from human life. By no other arrangement could our duties, our happiness, our mental and bodily perfections, have been bound together in one harmonious and consistent system. Let us compare the art of medicine, under this aspect, with those of navigation and agriculture. Had man been furnished by the Creator with wings, by which he could have traversed all seas and oceans, so as to supersede the use of ships, where would have been that hardihood of character, and all those ingenious devices which have called forth the active energies and deep researches of the human mind? If, contrary to the actual institutions of Providence, the life of man had been sustained by the spontaneous productions of nature, instead of the products of industry, neither the faculties of the mind nor the powers of the body could ever have been developed: man would have been little superior to the brutes; his active and inventive energies would have lain asleep for ever; there would have been no room for the talents exercised in the procuring of food, raiment, and shelter, nor in commercial intercourse; all the mutual and endearing ties and dependences of social and civilized life, all trades, professions, arts, and sciences, whether ministering to accommodation or elegance, constituting man's greatest felicity, whether as objects of pursuit or enjoyment, would have been unknown and untasted.

"It is obvious that this reasoning, being founded on a general law of nature, must apply equally to medicine. In a probationary existence, it was necessary that man should be tried, not only by pain and sickness, but by the difficulties of remedying them, as exercises of virtue and ingenuity. Why should the road to medical relief lie through fewer and slighter struggles and dangers, than those of navigation and agriculture? But the subject is more concisely and emphatically illustrated by the philosophical poet, than by any amplitude of illustration, or farther multiplicity of words, which I could employ:

———Pater ipse colendi (*medendi*),

Haud facilem esse viam voluit, primusque per artem,

Movit agros, (*ægros*,) curis acuens mortalia corda."

---

*A Treatise on the Nature and Treatment of Scrophula; describing its Connection with Diseases of the Spine, Joints, Eyes, Glands, &c. &c. &c. Founded on an Essay to which the Jacksonian Prize, for the Year* 1818, *was adjudged by the Royal College of Surgeons. To which is added, a brief Account of the Ophthalmia so long prevalent in Christ's Hospital.* By Eusebius Arthur Lloyd, Member of the Royal College of Surgeons in London; Senior Surgeon to the General Dispensary, Aldersgate-street; and late House-Surgeon to St. Bartholomew's Hospital. 8vo. pp. 330. London: J. Anderson. 1821.

The bibliology of scrofulous diseases is now nearly as extensive as that of consumption and stomach complaints; and, although the author before us asserts that "the disease [scrofula] has never yet been subjected to any particular and scientific investigation,"* he must mean within the circle of his own knowledge; for there is scarcely another complaint of the chronic kind that has had more monographers than the one he has undertaken to write upon. Without mentioning Hamilton's *Observations on Scrofulous Affections;* Nisbet's *Inquiry into the History, &c. of Scrofula;* Beddoes' *Essay on Scrofula*, in his *Hygeia;* Carmichael's *Essay on the Nature and Cure of Scrofula;* Russell's *Treatise on Scrofula;* Goodlad's *Essay on the Absorbent System*, and many more, one or two of which are named and quoted by Mr. Lloyd himself; we would wish to remind him of the numerous publications on the same subject which have appeared in other parts of Europe, particularly of those of Weber, Hufeland, Ritter, Pujol, Capelle, Hebreard, Baumes; all of which writers have treated the subject of scrofula in "a particular and scientific" manner within the last twenty years. We are not, however, sorry to see another labourer in this vast field of inquiry; and, when the ample opportunities which Mr. Lloyd has enjoyed of conducting a fresh investigation into the nature and causes of scrofula are taken into consideration, few there will be who will not be disposed to welcome a professed work on that subject from his pen.

"I have had frequent opportunities of examining by dissection the morbid parts, when they have been separated from the body by operation, as well as the bodies of those who have fallen a victim to the

---

* Introduction, pp. 1—2.

disease. I have also had, for many years, under my inspection patients labouring under this disease in all its various forms, and in many cases have been so fortunate as to be able to trace them from their commencement to their termination."

The public must not, however, be hurried away, in their judgment on the merits of the present performance, by the mere consideration of those favourable circumstances in which the author was placed, and which he has detailed in the above extract; nor by the more honourable fact, that Mr. Lloyd obtained the Jacksonian prize from the Royal College of Surgeons for the substance of this very essay. It is the mode in which an individual has availed himself of any advantageous opportunities he may have had for the promotion of science, and not the opportunities themselves, by which we should decide of his claims to public approbation; and, as to the reward he has already received at the hands of a most respectable body of medical men, we cannot forget that a similar one was awarded to another essay (Mr. Goodlad's,) some years before, in which the subject of scrofula had been treated on different principles; nor can we attach much importance to these sort of academical distinctions, when we reflect that another body corporate, of as great respectability as the College of Surgeons, once proposed the following question on the subject under consideration: "*An strumæ vel scrofulæ hoc etiam seculo, curari possint solo attactu regum Galliæ et Angliæ?*"

We shall now proceed to give as clear an account of Mr. Lloyd's performance as we can, offering from time to time such critical remarks as the subject-matter may suggest, in the same spirit in which the author himself has declared that he has spoken of the opinions of others,—"without any intention to give offence; and trusting, therefore, that none will be taken at the free expression of *our* opinion."*

The work is inscribed to Mr. Abernethy, whose great and constant kindness to the author, while a pupil in St. Bartholomew's Hospital, naturally called forth this token of gratitude. There were, moreover, two other motives which in this case influenced the author in selecting the object of his dedication:—"a wish to conciliate the public by an apparent union between himself and *Mr. Abernethy*;" and, lastly, a desire to maintain by facts and arguments *(verbis et armis,)* the truth of those principles which have been established by the same distinguished individual, concerning the dependence of local diseases on a general disorder of the system, and particularly on disorder of the digestive organs.

We may then, even *in limine*, assume the following as a

---

* See Preface, p. xii.

proposition of the author,—that *scrofula is a disease dependent on disorder of the digestive organs;* a point which Dr. R. Carmichael, of Dublin, endeavoured successfully to establish long before Mr. Lloyd, in his " Essay on the Nature and Cure of Scrofula, and a Demonstration of its Origin from Disorders of the Digestive Organs."

In the Preface, we are informed that one of the objects of Mr. Lloyd's was to make the principles of Mr. Abernethy, on which the treatment for scrofula is founded, better understood by those *out* of the profession; and that the great aim of the work is " to show that scrofulous diseases are, in general, to be removed by attention to the general health of the patient; and that the disease itself is to be considered the secondary, not the primary, object of attention."

Mr. Lloyd's book is divided into two parts. The first, extending to forty-eight pages, contains the author's general views on scrofula; its character and symptoms; its causes and treatment. The second part takes a wider range, and embraces considerations, practical as well as doctrinal, on local affections, the effect of scrofula.

I. *Characters and Symptoms of Scrofula.*—Authors in general, Mr. Lloyd thinks, have fallen into the error of describing the state of a patient labouring under scrofula, after the disease had given local evidence of its existence; for they enumerate among the symptoms of a scrofulous diathesis, or which only denote a tendency to scrofula, " a thickened, chapped upper lip; tumescence and redness of the tarsi, with weakness of the eyes in general; tumid belly; and enlargement of the lymphatic glands, particularly those of the neck." They ought, on the contrary, says Mr. Lloyd, to have informed us of the temperament or habit of body of the patient antecedent to the existence of any decisive evidence of the disease. We had therefore expected, in the book before us, a different description from that of any other author,—such a description as would enable us to become acquainted " with the nature of the disease in its first and earliest stage; to anticipate its march; and to prevent it, by every possible means, from obtaining a 'local habitation' in the body of any individual." In this expectation we were, however, disappointed; for, when we came to read that part of Mr. Lloyd's book which he considers as giving an account of the symptoms " indicative only of a disposition," we found it to embrace, more or less, the same symptoms mentioned by the authors he condemns; symptoms which do not describe a forth-coming disease, but a disease already settled and developed, as our readers will see from a perusal of the following extract:

" Scrofula oftener affects children than persons of any other age, though no age is altogether exempt from it. It appears, however, that the susceptibility which different parts have to it, is altered by age: thus, in children, the upper lip, eyes, glands of the neck, and those of the mesentery, are generally the parts first affected; the lungs, bones, and other parts, being subsequently attacked. It happens sometimes, too, in children, that small lumps form under the skin in various parts of the body, which suppurate, ulcerate, and pursue the same course with scrofulous abscesses in general. These we consider as scrofulous, from the nature of the matter they contain, and from their co-existing with other affections evidently of this description, and from other circumstances which will be mentioned in their proper place."

If we comprehend rightly Mr. Lloyd's meaning, it comes to this—that, if a child or an adult, enjoying an apparently perfect health, having neither "small lumps" under the skin, nor "a species of warts" about the face and neck, nor thick lips, nor a thick nose, nor an enlargement of the lymphatic glands, nor a tumescence and redness of the tarsi, nor a tumid belly, be presented to him, he (Mr. Lloyd) will be able to tell whether such a child or adult is likely to be affected by scrofula: "and will anticipate its march, and prevent it from obtaining a *local habitation.*" That the author himself may be able to effect this, we are by no means disposed to doubt; but, that he has imparted to us the same faculty by his description, is what we are not prepared to admit. Let the reader judge.

"The appearance of the countenance is really that of delicacy and langour; though, to the common observer, from the fulness, the peculiar smoothness, and the beautiful colour of the cheeks, it is often that of the highest and most complete health. If, however, it be attentively observed, it will be found that the cheeks, though full and tumid, are softer and more flaccid than is natural to health; and that, instead of being fixed and firm, they hang as it were loose on the face. There are, nevertheless, persons who have the greatest tendency to scrofula, in whom none of these apparent signs of health exist," [and he should say, where these apparent signs exist, who never afterwards exhibit symptoms of scrofula,] "but whose complexions are peculiarly dull and pallid; in these, the appearance of delicacy and langour is even greater than in the former. In the former state, the lips generally partake of the fulness of the face, and are of a beautiful red; while in the latter they are pale. It often happens, too, that the parts about the mouth are of a peculiar dull pallid hue. There is also a remarkable appearance about the eyes, which cannot be accurately described; but the conjunctiva is particularly free from blood-vessels, the pupil is generally much dilated, and the upper eye-lid drops more than natural; to which, perhaps, as well as to the delicate state of habit, the dilatation of the pupil may be in some measure owing."

With regard to the distinction commonly made between children of fair complexion and light hair and eyes, as more liable to scrofula, and those of quite a different external character, our author contends that it is not founded on legitimate grounds. The extensive investigation he has given to this subject, and his opportunities of examining numerous children in some of the largest charity-schools in London, authorize Mr. Lloyd to entertain a very different opinion, and incline him to say that "three-fourths of the children in this country have not what any impartial person would call fair complexions and light hair and eyes." If this be correct, then what remains for us to judge by as to the *probable coming-on of scrofula* in an healthy individual?

II. *Causes of Scrofula.*—Every day's experience convinces Mr. Lloyd that the local diseases called scrofulous arise from something wrong in the general health, sometimes co-existent with birth, and at other times entirely produced by improper attention to diet, to the alimentary viscera, and other occasional or accidental causes. This is intelligible as far as cause and effect; not so with regard to the *modus operandi* of the cause producing the effect. We confess we cannot look upon the explanation offered by the author as at all convincing, namely, "that certain derangement of the vital functions, capable of being produced by a variety of causes, may effect such an alteration in the natural actions of a part, as to impair its functions, and give rise to that change of structure which we term a scrofulous disease."

Were it so, how comes it that every deranged stomach does not give rise to scrofula? Mr. Lloyd may argue, that the deranged stomach must "affect *an alteration in the natural actions of a part*, so as to impair its functions, ere it can give rise to a scrofulous disease;" but, in that case, it will be incumbent upon him to demonstrate how such an *alteration in the natural actions of a part* as he alludes to, is brought about; and what sort of *deterioration of its functions* must that be which will give rise to scrofula rather than to any other disease. Until he have done this, the doctrine of scrofulous diseases is just where it was, before either Mr. Lloyd or Dr. Carmichael applied Mr. Abernethy's principles as a formula for solving this complicated equation. We know that the majority of our readers will side with us when we advance, that, in merely stating that A (the stomach,) produces an alteration in the natural actions, and impairs the functions, of B (another part of the body,) and gives rise to a subsequent effect (scrofula,) Mr. Lloyd has not thrown a clearer light on the etiology of that disease, than the *humorists* have done, with their virus,—the *chemio-physicians*, with their degenerated fluids, their alkaline and their acid principles,—or the *vitalists*, with their vascular debility.

Mr. Lloyd, in illustration of his position, that disorder of the digestive organs is capable of deranging the whole system, relates several curious cases of patients, who were recovering from various complaints, when, by an accidental derangement of the stomach, a stop was put to the progress of their recovery; and, among others, he tells us of one unfortunate patient, who forfeited his life in consequence of eating a large plum-pudding: but he has not related a single instance in which the eating of a plum-pudding has deranged the stomach so as to *alter the natural actions of a distinct part of the body, impair its functions, and produce scrofula*; yet nothing short of such a demonstration can be admitted, before we adopt Mr. Lloyd's etiology.

We, therefore, consider this part of the history of scrofulous diseases not to have benefitted by our author's publication.

III. *Treatment of the Disease.*—If we adopt the theoretical views of Mr. Lloyd as to the nature of scrofula, it follows that, "in the prevention and cure of the disease, our attention must be principally directed to the digestive organ: in the prevention, by avoiding all those causes which tend to disturb their functions; and in the cure, by tranquillizing irritation, and by restoring their healthy actions."

The means of prevention consist in warm clothing, the selection of a favourable climate, an appropriate diet, and a careful evitation of baneful passions of the mind, &c.

We select the following case, to prove the benefit which may be derived from warm clothing in the prevention of this disease.

"A young woman of very delicate health, who had had for several years the lymphatic glands of her neck diseased, and suppurating one after the other, and leaving ulcers difficult to heal, which were evidently of a scrofulous nature, was attacked with pain in her chest and cough, which were very obstinate, and resisted all the usual remedies. After this, when it was believed that her cough was incurable, and that she was in a decline, she was recommended to clothe herself from head to foot in flannel; which she did. From that time her cough began to get better, and in a few months was quite well; as were all the swellings and ulcers in her neck, which had for some years resisted all the medical remedies that had been employed. No new medical treatment was made use of during this period, and all the medicines she took were linctuses for her cough, and occasional doses of opening medicine. It is worthy of remark that, till she had recourse to additional clothing, she was particularly susceptible to colds, getting catarrh, sore throat, or inflamed eyes, on every trifling exposure to colder air than usual."

With regard to the treatment, properly speaking, our author very judiciously sets out by asserting, that no medicine we are acquainted with possesses any specific power over the disease. Mr. Lloyd has repeatedly seen soda, conium, cinchona, mercury, muriate of lime and barytes, steel, and the different mineral waters, fail, even without producing any visible effect on the complaint; though he acknowledges, at the same time, that patients have recovered, on taking some one of those medicines, after suffering for a considerable time in spite of the administration of various other medicaments.

The author, having declared that an improper attention to diet is one of the most frequent causes of disorder of the digestive organs,* (a truth which there can be no difficulty in believing,) naturally directs us to be particularly attentive to the diet of our patient in scrofulous diseases.

"To attempt to describe the particular diet that scrofulous patients should live on, would be absurd, but the principles which should guide our choice may be stated in a few words. We should avoid every kind of stimulating diet, but it should be good and nourishing,—as milk, eggs, arrow-root, meat-jellies, &c.; but of none of these more should be eaten at once than can be easily digested; and the meals should be taken at regular periods, and after regular intervals, for nothing can be worse than living irregularly: perhaps, fasting for the whole day, and then eating one immense meal, as the natural consequence of it, must be to excite the whole system to a state of fever, besides the immediate bad effect that it has on the stomach and bowels. All stimulating liquids, too, should be avoided; and, as hunger and thirst are, as Mr. Abernethy states in his Lectures, incompatible sensations, eating and drinking should not be indulged in at the same time, but the same regularity should be observed in the one as in the other."

The above observations apply to the simple state of a scrofulous constitution; but, where the disease has made great progress, all the natural functions are disturbed and hectic induced —a generous diet and stimulating drinks will be found useful.

The next point of importance in the treatment, is the keeping the bowels regular, and the hepatic secretions natural.

"The means that I would make use of for the accomplishment of this point, are the following. If the patient be an adult, and the bowels obstinately confined, I would give him five grains of the blue-pill every night, and half a pint of dec. sarsæ. co. twice a-day; and, if by a certain time of the day the bowels had not been open, I would give some opening medicine, and repeat it at certain periods until it operated. This plan I should pursue till the bowels become regular; and then, to prevent their relapsing into the same state, I would continue the exhibition of alterative doses of mercury for an indefinite time; and the form I would make use of would be the compound calomel pill, in doses of five grains every other night. If it be a child that is affected with this disease, the same principles would guide my practice; but, as the constitution, as well as the age, of children who become affected with this disease may be very different, it is impossible

* Page 37.

to know at once what medicine will be applicable to each particular case. Indeed, every one must have observed that the same medicine may act very differently on children of even the same age; and that what purges one violently will have no effect on another. We should, too, be very careful not to give violent purges; and we should particularly avoid large purgative doses of calomel, as I am convinced they often produce more general irritation than the evacuation they occasion from the bowels is able to relieve; and that they often so much weaken the stomach, that it is a very long time before it is able to recover its natural powers. Our object, therefore, in prescribing medicines, should be to procure a proper emptying of the bowels daily, and a healthy condition of the dejections. This, I admit, is often a difficult point to obtain, but by proper management we may generally succeed. Any of the mild purgative medicines may be employed for this purpose, and, if one does not appear to have the proper effect, we should desist from its use, and substitute another; but we may derive the greatest assistance from exhibiting alterative doses of calomel at the same time. The dose should be varied from half to one grain, according to the age and habit of the child, and repeated twice or thrice a-week. Sometimes, in particular states of the stomach and bowels, it is better to combine the calomel with the purgative, and at other times they act better given separately. The very great influence which evacuations from the bowels have over the rest of the body, cannot be denied by any impartial observer: it is, therefore, certain that, by increasing or diminishing them, we are able to produce a decided effect on the whole, or, as I have proved before, on a particular part of the body. Thus, if there is much general irritation, or local irritation and inflammation, by increasing the intestinal evacuation, taking care however not to irritate the bowels, we may very much relieve both the one and the other. The importance, therefore, of attention to the state of the bowels is obvious."

Small doses of soda should be exhibited when a great degree of acidity prevails in the stomach; else, by remaining there, or passing into the bowels, it may do mischief. When there is a weak stomach, with loss of appetite, cinchona, steel, and the mineral acids, will be found of the greatest service. Of cold sea-bathing, Mr. Lloyd has no good opinion. He has seen it fail in removing the complaint in almost all its various forms; nor does he know a single instance in which he could fairly say that it had removed it. It may be useful in checking a disposition to the disease; but it certainly has no power of discussing scrofulous humours, or of healing scrofulous ulcers.

With regard to a residence at the sea-side, Mr. Lloyd insists on scrofulous patients not remaining longer than four, or at most five, months; though the *wherefore* is not apparent, since he immediately adds, "that generally he does not believe there is any material benefit to be derived by sending them there."

Good air and exercise are conducive to the cure of this disease: the warm sea-bathing Mr. Lloyd thinks injurious to the general health.

Of the inefficacy of sea-bathing in the cure of scrofulous diseases of the spine, hip, or knees, Mr. Lloyd saw a corroborating example, of recent occurrence, which he relates not quite in the spirit of charitable forbearance towards a fellow practitioner.

"It is the case of a little boy who had disease of the right hip-joint, and about whom I was consulted a little more than two years ago. At this time I made an issue behind the great trochanter, and kept the child lying down, and the case went on as favourably as possible. A few weeks after, however, I was taken ill, and therefore unable to attend the case. Another surgeon was therefore consulted, and he at once recommended that the child should be sent to the sea-side; which was immediately done, and he continued there till very lately. Since his return, I have seen him; and I certainly never saw a much more horrible state of deformity, or a case more fully proving the inutility of sea-bathing in these diseases. The spine is much curved; the right hip forms an immense projection; the thigh is dislocated, and much bent on the body; the knee contracted, and the leg bent on the thigh, and the limb so much shortened that, with the foot bent downwards, the toe will only just reach the ground; and, what is still worse than all, the disease is still going on. Now, I am fully convinced that the whole of this deformity, except perhaps a trifling shortening of the limb, is entirely owing to improper treatment, to want of rest, and to the body not being kept in a proper position. This child, however, must remain a complete cripple, and an object of pity, as long as he lives. I believe, however, that the disease may now be put a stop to, and some part of the deformity removed."

Mr. Lloyd terminates the first part of his book with the following conclusions, *fairly* drawn (as he says) from his foregoing observations; upon which we forbear making any remark.

"First. That scrofula is hereditary, but that the tendency to it may exist without its being called into action.

"Secondly. That a disposition to it may be acquired where there is no hereditary tendency.

"Thirdly. That all those causes which tend to derange the natural actions of the body are capable of inducing scrofula, and that it always is a constitutional disease,—that is, never depending upon local causes only.

"Fourthly. That the disease may generally be prevented, by avoiding all those causes which have a direct influence in disturbing the general health.

"And fifthly. That the disease is only to be cured by avoiding all sources of irritation, and by restoring the natural and healthy functions of the digestive organs."

The second part of Mr. Lloyd's book treats, as we have already observed, of the local effects induced by scrofula. There is no structure, nor particular part of the body, which may not be attacked by scrofulous diseases, though some parts are more obnoxious to it than others. Those of which the author treats in detail are the following.

I. *Scrofulous Affections of the lymphatic and some other Glands.*—A scrofulous gland, in an early stage of the disease, is simply enlarged. If the disease is not put a stop to, the whole structure of the gland becomes altered and destroyed, and new matter is gradually deposited. At other times, the gland is converted into a much softer matter, and, when cut into, appears to be composed of two kinds of substance,—the one resembling curd, and the other being softer, less opaque, and of a yellow colour. Inflammation of the surrounding parts comes on after a time; suppuration follows, and ulceration in one or more places occur. The gland, however, Mr. Lloyd contends, is not included in the suppuration; and the abscesses formed around it are known to be scrofulous from the matter which they contain, which is a wheyish fluid mixed with a curd-like matter, with an occasional appearance of what seems to be tolerably good pus.

Several glands often enlarge at the same time, and continue distinct till they have each acquired a very large size; but, at length coalescing, form one immense tumor, which sometimes presses so on one side of the larynx and pharynx as to impede respiration and deglutition. These tumors, even when they have obtained this size, may subside entirely without the formation of matter; of which fact Mr. Lloyd gives the following example:

"The first is the case of a young man, a patient in St. Bartholomew's Hospital, aged twenty-one years, who had several glands of the right side of his neck enlarged for above two years, without their producing much inconvenience; but, his health becoming very much deranged, they increased in size, coalesced, and formed a large tumor, in which there was no fluctuation, which produced great difficulty of breathing and of deglutition, and was attended by violent head-aches. By attending to his general health in the manner that has been recommended, by applying six leeches twice, and a bread and water poultice night and morning, the tumor diminished at least two-thirds in the space of ten weeks; and subsequently entirely disappeared."

Besides the lymphatic glands of the neck, there are those of other parts of the body that are liable to be affected.

"It is very common to have a gland enlarge, and form an abscess about the middle of the tibia; just behind the inner condyle of the femur; also just above the inner condyle of the humerus; on the inside of the ulna, near the olecranon; and above the middle of that bone: above the clavicle, too, is not an uncommon situation. The glands of the groin are frequently affected."

Scrofulous abscesses will sometimes form in the parotid glands. The author relates the case of a young woman who had a scrofulous abscess in her right parotid gland, and who eventually recovered. The thyroid as well as the prostate gland are occasionally the seat of scrofulous abscesses; and, in fact, abscesses of a similar nature may form in every part of the body, independently of the glands. Mr. Lloyd thinks that, in this case, they suppurate more quickly, and that not unfrequently the fluid will entirely disappear. Our own experience does not warrant the above conclusions. Among other cases of the kind last mentioned, we recollect attending one with Mr. Brodie,—a child of a lady of rank,—where the swelling, situated on the centre of the curve of the fourth and fifth ribs, remained of the same size, and with the same degree of tension and fluctuation, for several weeks, notwithstanding repeated applications, intended to discuss in the first instance, and next to promote suppuration. In this case, the muriate of barytes, the blue pill, and afterwards bark, played a conspicuous part in restoring to health the general constitution. The abscess was eventually opened.

We were rather surprised, after reading so much about the simplicity of Mr. Lloyd's views respecting the etiology of scrofula, to find him employing, in this part of his book, a language like this,—that "the uniformity of the effects of scrofula fully proves its title to be termed a specific disease, and its action to be an action *sui generis*, the peculiar nature of which it would be idle and absurd to discuss." Thus has Mr. Lloyd been reduced to use the identical language of his predecessors, all of whom he indiscriminately condemns for that, without which he himself cannot explain the nature and mode of action of the disease he has undertaken to descant upon. Yet, because Mr. Goodlad has not chosen to call in the aid of this something having an action *sui generis*, to explain the phenomena of scrofulous disorders, our author, who ought to have commended him for his approach to simplicity, finds fault with him for it; nay, accuses him downright of never having cultivated morbid anatomy. We really cannot comprehend so much apparent contradiction.

*Treatment.*—The less that is done locally to the glands, when simply enlarged and in an indolent state, the better. Proper constitutional remedies should be had recourse to. The part may be bathed with salt and water. In the more advanced stages, irritation must be allayed by soothing applications, and by leeches and warm emollient poultices. Mr. Lloyd gives a proper indication for the application of blisters; but, with regard to opening a scrofulous abscess, he states that no definite rule can be laid down.

"When any accidental occurrence, as a blow, or the health becoming suddenly much disturbed, excites those parts which had been for a long time in an indolent state to a state of active inflammation, and matter suddenly forms, producing tension and pain; or if the abscess be situated in the vicinity of a bone, as the tibia or ulna, it undoubtedly should be opened as soon as possible. But, if none of these circumstances occur, if there be neither tension nor pain, although the whole skin covering the abscess be discoloured, and the fluctuation be evident and distinct, and the whole tumor reduced to a state of softness and flaccidity, I believe it is of very little consequence whether a puncture be made or not."

II.—*Scrofulous Ulcers.*—"The only characteristics of a scrofulous ulcer, that can be depended on, are its occurring after a scrofulous abscess, the peculiar dull red or purple colour of its edges, its remaining indolent for a great length of time, neither increasing nor diminishing in size, and its being attended by the peculiar state of health which has been already described."

"I know of no specific remedy for a scrofulous ulcer; nor can we rationally hope to find one for any local disease depending upon a peculiar state of the general health."

"Of all the variety of local applications, I know of none that agree with scrofulous ulcers better than those that are slightly astringent; and what is called the diluted citron ointment is, in my opinion, the best. Under certain circumstances, it may be combined with a small proportion of the ceratum resinæ, or of the ungt. zinci. Sometimes, however, the different preparations of lead, and indeed all other local applications, appear to be useful; while in other cases they evidently produce no effect at all."

Mr. Lloyd afterwards details two cases, under the division of *scrofulous ulcers*, which we are at a loss to discover what they are intended to illustrate, as no ulcer was present in either of them. Many other cases, more or less interesting, but all of them detailed with much candour, are given in this subdivision of the work; to which we earnestly request the attention of the readers.

III. *Scrofulous Affections of the Female Breasts.*—"This affection generally commences by the formation of a hard moveable lump in some part of the breast. This gradually increases in size, and coalesces with the surrounding parts, which become tender, inflame, and form successive abscesses, which leave small ulcerated openings; and these are sometimes very difficult to heal. It also occasionally happens that the whole skin of the under part of the breast, where these abscesses are generally seated, becomes discoloured and diseased; that the swelling increases; and that a considerable degree of constitutional irritation ensues, which is seldom relieved, more than in a very slight degree, by either general or local bleedings. Under these circumstances I have found the insertion of a seton highly serviceable."

We shall only quote a case in illustration.

"A young woman, aged twenty, had a small hard lump formed in the under part of her breast, which gradually enlarged till it was lost in the surrounding parts; became painful, inflamed, and formed an abscess, which discharged about three ounces of a scrofulous matter. A small ulcer was left: and the parts, which were covered with a bread and water poultice, became in a quiet state. Her health had been for many months in an indifferent state, and her catamenia were suppressed. The constitutional treatment that was adopted consisted principally in keeping her bowels open, and in attention to her diet.

"She, however, did not materially improve in health, and abscesses continued to form successively; the swelling of the breast rather increased, and was attended with great pain. I therefore took from her arm twelve ounces of blood, and for the first two days the pain was less, and her pulse more tranquil; but, the pain again returning, I bled her again to the same extent, but without benefit. Leeches were also applied repeatedly to her breast, but with the same success. She now became very impatient; I therefore made a seton under the breast, at a little distance from it, and continued the treatment that had been previously adopted. From this period a complete change appeared to take place in her, as both her health and her breast began rapidly to amend; so that in less than two months the breast was reduced to its natural size, her catamenia re-appeared, and her health was very good: and in less than another month I removed the seton, as the ulcer had healed, and only a slight hardness remained."

IV. *Disease of the Testicle.*—This part of the human body is certainly subject to scrofula, both in the child and in the adult. In the adult, it gradually enlarges, and the progress of the disease is very similar to that of the same affection of the absorbent glands. The enlargement is of two kinds. In the first there is interstitial deposition, or simple expansion of the natural structure of the gland. In the second, the natural structure of the gland becomes entirely absorbed, and a matter, similar to what is seen in the cancelli of scrofulous bones, of the consistence of cheese, and of a yellowish-white or greenish-yellow colour, is deposited in its place. The coats of the testicle, in both species of enlargement, continue for some time healthy and unaltered. There are several other points connected with this part of the inquiry, which Mr. Lloyd has detailed, we think, in a satisfactory manner, and which deserve consideration.

*Treatment.*—In the first stage, the use of a suspensory, to take off the increased weight of the testicle, and the application of a bread poultice and cooling washes. In the second stage, poultices again, and leeches. The constitutional treatment may consist of sedatives and mild purgatives. Mr. Lloyd is convinced that many of the chronic enlargements of the testicles, which we so often meet with in practice, are of a scrofulous nature, and that the generality of them are certainly curable. He therefore does not advocate the practice of castration, notwithstanding the high authority of Pott. Among the numerous cases of diseased testicles which occurred in Mr. Abernethy's practice, during the author's long and active attendance at St. Bartholomew's Hospital, only one required castration; and that was a case of fungus hæmatodes. The epidermis is sometimes affected with scrofula, without the testicle partaking in the slightest degree of the disease. Mr. Abernethy possesses a preparation of this modification of the disease.

V. *Scrofulous Affection of the Prostate Gland.*—Mr. Lloyd has not met with any instance of the prostate gland converted into scrofulous matter, but has seen repeatedly cases of fleshy enlargement of that gland. They occurred generally in young men of a scrofulous habit.

"These cases generally get well by tranquillizing the constitutional disturbance, and by making use of soothing local applications. A bougie should also be occasionally passed, even though there should be no stricture at the time; as, from the state of the prostate and the high degree of irritation in the urethra, there will always be a great disposition to form stricture, which the bougie will prevent, as well as tend to diminish the particular irritation in the urethra, and in the prostate itself. It will also, more effectually than any other means, remove that most troublesome symptom, the frequent desire of making water, which so often attends this complaint. The too frequent introduction of bougies, however, is undoubtedly injurious. Suppositories of opium and other medicines have been recommended to remove spasm and irritation from the neck of the bladder, and in common cases, I believe, they sometimes do good; but, when the irritation depends upon a diseased state of the prostate, particularly of the description now under consideration, I should never advise their use. I have frequently tried them in these cases, and I have oftener seen them aggravate than allay irritation. I never either have seen any of that class of medicines called discutients have the slightest effect in reducing this enlargement of the prostate gland; but, as I have stated before, it will generally subside if the state of the constitution be improved, and if no other means be made use of locally but those which relieve irritation."

The author next notices the scrofulous abscesses of the prostate gland, and the circumstance of the matter contained within them making its way into the cavity of the bladder. He also states, to have seen the vesiculæ seminales affected with scrofula, and filled with a cheesy matter.

VI.—*Scrofulous Affections of the Bones and Joints.*—The description, as also the details of the treatment, of this modification of scrofula, form by far the best, as well as the most useful, part of Mr. Lloyd's book. They deserve unqualified commendation, and we crave our readers' earnest attention to them. There is much of practical and experimental wisdom in this subdivision of the work, which stamps it at once as a classical book.

VIII. *Scrofulous Disease of the Hip-joint.*—The progress of scrofula, and the alteration which it occasions in the structure of the hip-joint, so much resemble what occurs in other joints under the same circumstances, that Mr. Lloyd thinks it unnecessary to extend far his observations on this part of the subject.

"Scrofulous disease in the hip-joint, as in all the other joints, comes on so gradually and insidiously, that it is scarcely possible to discover it till the morbid alteration of the bone has gone on to a considerable extent. The first indications that we have of this disease, if we except the disorder of the general health, are, that the patient is a little lame, and walks on his toe, and that he complains of being tired much sooner than he would if he were in health. This state will often exist for several months without the patient feeling pain; but at length the lameness increases, and the affected limb is never carried so far forward in progression as the opposite one, and shooting pains are occasionally felt in the joint and other parts of the limb. Upon examination, the muscles of the thigh and nates of the affected side are found wasted; and, as the disease advances, the wasting of the muscles increases, and the limb becomes evidently smaller than the opposite one, and the nates of that side appear widened and flattened. If pressure be now made over the joint, behind the great trochanter or in the groin, it occasions pain, and the bone about the trochanter often feels as if it were thickened and enlarged."

It is not uncommon for the glands of the groin to take on the scrofulous action, to enlarge, and suppurate, as one of the first symptoms of the joint of the hip being diseased.

*Treatment.*—The constitutional treatment of scrofulous disease of the hip-joint, of course, differs in no respect from that recommended in all other scrofulous diseases. In the local treatment, on the contrary, there are certain minutiæ to be attended to, arising from the nature of the part affected.

"Directly it is ascertained that the hip-joint is diseased, it is always absolutely necessary to strictly enjoin the most perfect rest, as, without attention to this point, all the remedies we may employ will generally fail in arresting the progress of the disease. As, too, in all the varieties of hip disease, there is invariably a great disposition in the thigh to become bent upon the body; in the leg, to become bent upon the thigh; and in the spine, to become awry; a mere state of rest is not all that is requisite, but it is also important that we attend to the position of our patient. The patient, therefore, should not only be constantly confined to bed, but should also lie in an horizontal position; the lower extremities should be kept constantly straight, and the trunk of the body should not be allowed to incline either to one side or to the other, so that the heels shall be as near as possible in the same line."

Rest will do much to prevent irritation. The abstraction of blood from the part, by cupping or leeches, does not appear to the author to be attended with much, if any, good in disease of the hip. The early use of counter-irritants, Mr. Lloyd has not recommended in scrofulous diseases of the other joints, because they seem rather to increase the swelling of the parts, and consequently to promote the formation of abscesses. In the disease of the hip-joint, however, their early use is highly serviceable. Mr. Lloyd mentions this as a fact, and not with reference to any particular theory. The various modes of making counter-irritation, such as the actual cautery, moxa, issues, setons, scarification, perpetual blisters, tartar-emetic ointment, &c. are next considered, though superficially.

The author states, that, if the plan of treatment recommended in the preceding pages be adopted from an early period of the disease, and the *vital organs are as yet sound*, he feels no doubt about the successful termination of the case.

Two cases of this disease, which proved fatal, with an appearance of the parts on dissection, we shall quote, as highly instructive.

"The first is the case of a poor boy, aged fourteen, who died of diseased hip, which had existed for nearly four years. The femur had become dislocated, and the limb much shortened; and abscesses had repeatedly formed in the joint, or in the parts surrounding it. At the time of his death, he was suffering from severe cough and profuse expectoration of matter. His abdomen was very large; and he had troublesome diarrhœa, which had continued for above a month. He had also a confirmed hectic.

"Upon examining the hip after his death, the whole joint was in such a confused mass of disease, that it was impossible to say in what part it commenced: however, upon examining the knee-joint, some clue was given to the nature of the disease. In this joint, the bones were much softened, and the cancelli filled with a cheesy matter. The cartilages were entire, but there was about half an ounce of synovia in the cavity of the joint. The soft parts were in a healthy state. Upon examining the abdomen, there were found nearly four quarts of serous fluid collected in its cavity; and the mesenteric glands were most extensively diseased, some being converted into a cheesy substance, while abscesses had formed in the place of others. Upon examining the lungs, they were found tuberculated, and vomicæ had formed in different parts of the right lung.

"The next case is very similar to the former. A poor boy, aged seventeen, died in May 1818, of hip disease, which had existed for nearly three years. The pain which had accompanied it had never been very severe. The head of the femur had become displaced, the limb was shortened, and abscesses had formed all round the joint, and left sinuses wherever they had burst. Several glands of the groin had also suppurated, and left scrofulous ulcers. At the time of his death, he had a great difficulty in breathing and a troublesome cough, though without much expectoration.

"Upon dissection, the head of the femur was found in great part destroyed, but what remained was resting on the dorsum of the ilium, which at that part was slightly carious. The acetabulum was full of lymph and pus, and the cartilages, at its edges, slightly eroded. The trochanter major was in part so soft you might easily cut through it, while the neck of the bone was hard and unaltered in structure. The lungs were tuberculated, and in the right lung there were two large abscesses containing complete scrofulous matter, mixed with a very little blood. The mesenteric glands were many of them much enlarged, and some of them partly converted into a cheese-like matter."

VIII. *Scrofulous Disease of the Spine.*—This is another very important subdivision of the work before us. The subject has lately attracted so much of the attention of the public and the profession at large, that our readers must feel anxious to learn the author's opinion on so interesting a subject. We cannot, however, in justice to Mr. Lloyd, do more than invite our readers to consult the book itself; for we apprehend we have already analyzed and extracted fully as much as a due consideration of the author's interest would admit, and to go beyond it might savour of dishonesty.

Mr. Lloyd considers the scrofulous disease of the spine under the heads of "angular curvature" and "lateral curvature." The symptoms produced by each sort of curvature are minutely detailed; and next, the individual treatment of each. There is also an account of the symptoms indicative of a disposition to curvature, which merits attention.

Mr. Lloyd is in direct opposition to the principles broached, in this Journal, by Dr. E. Harrison. He supports the doctrine of Pott respecting the nature of spinal diseases, and avers, that dissection, which is, after all, the great test in these cases, shows the reality of the caries and absorption of the vertebræ in angular curvatures. The condition of the invertebral substance, or each layer, as they are termed, is extremely uncertain. Many interesting examples, in support of these facts, are reported by our indefatigable author.

Thus far we have followed Mr. Lloyd as a surgeon of no common talents, and one who seems to have profitted of the many favourable opportunities he has enjoyed, for the promotion of one of the most interesting branches of surgical investigation. When, however, he enters on the province of the physician, and undertakes to treat of scrofulous diseases of the viscera, all of which he debates in the course of twenty-eight pages only, we are inclined to exclaim, *Ne sutor ultra crepidam.* We have no hesitation in saying, that the physician will find nothing in this part of the work, by which he can improve his present knowledge of the diseased condition of visceral organs, labouring under the baneful influence of what has been termed a scrofulous diathesis. The few facts which Mr. Lloyd has added to our stock of morbid appearances in these cases, will not contribute, we apprehend, to throw any better light on the intricate question they are brought forward to illustrate.

The book concludes with a section on "scrofulous ophthalmia;" and a curious account of the ophthalmia so long prevalent in Christ's Hospital.

We shall not sum up, after detailing so much at length the evidence by which our readers are to judge of Mr. Lloyd's book; but will leave them to decide on the extent of its merits, unbiassed by any observation of our own.

---

*A Manual for the Student of Anatomy; containing Rules for displaying the Structure of the Body, so as to exhibit the Elementary Views of Anatomy, and their Application to Pathology and Surgery.* By John Shaw; *being an Outline of the Demonstrations delivered by him to the Students in the School of Great Windmill-street.* 12mo. pp. 342. Burgess and Hill, London.

The opening of the new scholastic year for the students in medicine, has induced us to take an early notice of the present work. To those who are about to acquire the first rudiments of anatomy, Mr. Shaw's book will prove a valuable present. It will be a clear and sure guide to them; it will serve to smooth the path through the various difficulties, and often intricate, researches of anthropography; it will assist their memory in the storing-up of newly-learned facts; and, lastly, it will be found a very useful syllabus, and one of the best text-books for an anatomical class.

Of such a book, of course, it is unnecessary to give a minute analysis; but that which we cannot omit to give, is an account of the manner in which the work has been composed. By doing this, we shall, doubtlessly, excite a desire in the junior branch of the profession to possess the book; an object, in the accomplishment of which a reviewer should centre all his efforts; since it is thus that the best interests of science are promoted, when a work of merit is the subject of critical consideration.

In an Introduction, of six pages, the young student is put in possession of some general rules upon his entering on a course of dissections. Here the author inculcates the necessity of attending to the real end of anatomy, which does not consist in teaching *minutely*, but philosophically and practically, the organization of those parts, the recollection of which would be of use when the student is left to his own resources. With respect to the instruments required by young dissectors, Mr. Shaw has the following observations:

"The student will find that he requires several instruments, besides those generally put into the dissecting-case, to enable him to make some of the more difficult dissections. Thus, for example, he could not dissect the nerves of the spine, nor of the head, without a small saw, two or three chissels of different sizes, a small mallet, and the strong pincers (that are used to pull out nails); the knife (called a hacking-knife,) which is used by plumbers to cut lead, will also be found very convenient.* For the more minute dissections, he will require two small hooks and a sharp steel point. The etching-tools which are used by engravers are very useful, particularly if the points are bent a little, as we can then easily tear away the cellular membrane from the small nerves."†

To prevent the consequence of too close an attendance on dissections, and to remedy the bad effects of accidental punctures during the operation of dissecting, the suggestions of our author should not be neglected.

"I think I have observed, that it is necessary for students from the country to live a little *fuller*, while attending the dissecting-room, than they have been accustomed to do while in the country. If they do this, and at the same time take regular exercise, and attend to the state of their bowels, they will generally escape the bad consequences which occasionally occur from a cut on the finger.

"The best treatment for the inflamed lymphatics and the swollen arm, is to apply lint, soaked in the sugar-of-lead lotion and tincture of opium, to the arm; and to take calomel purges, and large doses of opium, with plenty of wine and porter."

The first object to which the student is directed to attend, who wishes to acquire elementary views of anatomy, is the dissection of the muscles of the abdomen, the process of which Mr. Shaw details with much clearness and precision; and, as it was scarcely possible to do this without, at the same time, referring to some of the most important surgical operations performed on this part of the body, the reader will not be surprised to find here a description of the parts connected with hernia, particularly the fasciæ, with some remarks on the changes which those parts undergo when hernia takes place. We were rather surprised to find Dr. Breschet's name totally omitted, in treating of the important subject of hernia, the anatomy of which that gentleman has very recently and greatly contributed to

* "All these things may be got at a carpenter's tool-shop; the chissels which are used for cutting iron are the best.

† "It is necessary to have one or two coarse cloths, to cover the parts which have been dissected; as they very quickly spoil when left exposed to the air."

illustrate. We cannot help observing, that, in speaking of his predecessors or contemporaries, Mr. Shaw has assumed a sort of dictatorial tone, which, we humbly submit, ill becomes a young teacher, and one whose name has not yet travelled far; though we venture to predict that it will do so, at no very distant period. Mr. Shaw is also, we think, too fond of insinuations, where he does not consider it safe to be explicit. In the Introduction, there are two or three instances of such insinuations, evidently directed against some teachers and dissectors, which savour strongly of bad taste, to say the least of them. Mr. Shaw should also learn to be more frugal in his commendation of the teacher whose assistant and relation he is known to be, and whose lauds, when chaunted so frequently by an interested party, may, in the eye of the malicious, appear to be dictated by partiality. On another point we beg to make only one observation. We are decidedly of opinion that the quaintness of style and affected construction of language, which Mr. Shaw has adopted, in evident imitation of the same master, will prove injurious to his future reputation. However well such a style and such a language may be thought, by that gentleman and Mr. Shaw, to suit the purpose of oral delivery, there is no doubt but that it ill becomes the dignity, as well as the simplicity, of written composition. It was well said, by the Edinburgh reviewer, on a recent occasion, when remarking on the numerous fustian-writers of the present day, that, if they will not condescend to the unstudied and familiar mode of communing with the public, they should, at least, have the art of concealing their *art*, and not obtrude the conviction that they are more anxious to display themselves than to inform their readers.

Mr. Shaw knows so well how highly we think of his talents, and how great are our expectations of his future professional exertions,—and we are so fully convinced that he has no need of meretricious adjuvants to take and maintain his proper and enviable station amongst us,—that we have been the less scrupulous in giving him, on the present occasion, a well-meant piece of advice.

We now proceed onwards with our account of his excellent work.

The parietes of the abdomen having been removed, the viscera of that cavity offer themselves to view. The observations respecting them are full of practical utility. Mr. Shaw treats afterwards of the dissection of the diaphragm and deep muscles of the abdomen; and gives the proper method of examining the minute structure of the several viscera, as well as of injecting and dissecting the arteries and veins of those viscera.

We wish we could spare room for Mr. Shaw's judicious remarks on "the manner of examining a body, to discover the seat of disease." His observations on the appearance which denote previous inflammation in the intestines, we must positively quote, though stinted for space in our reviewing department.

"It is a very common mistake to describe the loaded state of the vessels as an appearance denoting previous inflammation: the state of the true inflamed intestine is so distinct, that it can hardly be forgotten after it has been once seen. In the first stage, there are numerous small vessels seen upon the gut, like those on the eye in ophthalmia, with a suffusion around them; in the second stage, there is matter, or lymph, effused; and, in the more advanced state, adhesions are formed between the surfaces of the intestines. But there are many different kinds of peritonitis. In that which is called idiopathic, the peritoneum will be found coated with lymph; but, after inflammation in consequence of strangulated hernia, the substance of the intestine will appear more affected than the proper peritoneum."

The next great division of Mr. Shaw's book will be found very important: it relates to the male pelvis generally, and to the anatomy of the perineum in particular. The great question of lithotomy, of course, is fully considered. On this point there is a sort of *good-natured* note, which illustrates, more than any thing else, our observations on the style and composition of our author, as being an imitation of those of Mr. Bell.

"When discussing the operation of lithotomy with some of the young students in the dissecting-room, I have very often put this question, when the body is before them,—'What is your object in performing the operation of lithotomy?' Though this question is considered rather insulting, still it leads them to form a correct notion of one of the great principles of the operation, viz. to *cut low in the perineum*, that the extraction of the stone may not be obstructed by the narrow part of the bony arch."

How, in the name of good sense, can the insulting question, "What is your object in performing the operation of lithotomy?" lead pupils to form the correct notion that they must *cut low in the perineum*? Who is there that does not see in this apostrophe, and Mr. Shaw's description, in the same note, of the "pulling" and "dragging" of the stone by the forceps, the same spirit and taste which moved the pen of another author, when, on speaking of penetrating wounds of the chest, he began one of his chapters or paragraphs (we really do not recollect which), thus:—*If the midnight assassin plunges his stiletto*, &c. &c. as if any other miscreant's stiletto would not do for the purpose, or could not as fully illustrate the subject under consideration.

A few pages are dedicated to the dissection of the parts within the pelvis of the female. They are clear and accurate.

The muscles and ligaments of the extremities and joints are considered next, with various tables, showing the origin and insertion of the former, and the disposition of the latter.

The completion of the dissection of the *lower part* of the body, according to Mr. Shaw's plan, takes in an account of the arteries, veins, and lymphatics, and touches on the nerves of the thigh and leg. This compartment of Mr. Shaw's Manual contains, moreover, several very useful practical remarks for tying the external and internal iliac arteries, the gluteal and ischiatic, and the superficial femoral; as well as some observations on the anatomy of buboes and lumbar abscesses; and the dissection of the parts in the ham, when there is popliteal aneurism.

In the dissection of the *upper part* of the body, the student is successively led to a knowledge of the internal and external parts of it, by a methodical and very clear process. We have, first, a mode of dissecting the brain, so as to show each part and the origin of the nerves; and next, the manner of opening the spinal canal, and of examining the encephalic structure, with a view to discover the appearances of disease. Here Mr. Shaw adds some observations on the difficulty of deciding as to the cause of death, in supposed injuries of the brain.

The dissection of the thoracic viscera follows that of the muscles of the face, neck, and chest; and is succeeded by that of the muscles of the back, spine, jaw, ribs, &c. Here also we have some useful practical directions for discovering the seat of disease in the viscera of the thorax, and for distinguishing the morbid from the natural appearances. In speaking of the appearances which a lung will occasionally take up after long disease, so as to resemble the substance of the liver, Mr. Shaw says, that the French pathologists have called it "pulmo hepatizè." Mr. Shaw meant *poumon* hepatizè.

Many other miscellaneous subjects, of more or less importance, are treated of in succession by Mr. Shaw, towards the termination of his book, which we have not room to detail; and the book itself concludes with a description of the plans intended to illustrate what Mr. Shaw has called "the new arrangement of the nervous system," by Mr. Charles Bell. The outline of this new arrangement we shall give in Mr. Shaw's own words. The plans to which the author refers are two plates, which accompany the "Manual for the Student of Anatomy."

"The principal arrangement is this:—There is an obvious division of the medulla spinalis, corresponding to the cerebrum and cerebellum; every *regular nerve* has two roots, one from the anterior of these columns, and another from the posterior. Such are the fifth pair; the *suboccipital;* the *seven cervical;* the *twelve dorsal;* the *five lumbar;* and the *six sacral;* viz. thirty-two *perfect, regular,* or *double nerves.* These are laid down in the first plan. They are common to all animals, from the worm up to man; and are for the purposes of common sensation and motion, or volition. They run out laterally to the regular divisions of the body, and never take a course longitudinal to the body.

"For the sake of arrangement (although the term be not correct where every thing is perfect,) the remaining nerves are called *irregular nerves.* These are distinguished by a simple fasciculus, or single root; that is, a root from one column. These are *imperfect* in their origins, *irregular* in their distribution, and *deficient* in that symmetry which characterizes the first class. They are superadded to the original class, and correspond to the number and complication of the superadded organs. Of these, there are the third, fourth, and sixth, to the eye; the seventh, to the face; the ninth, to the tongue; the *glosso pharyngeal,* to the pharynx; the *vagus,* to the larynx, heart, lungs, and stomach; the *phrenic,* to the diaphragm; the *spinal accessory,* to the muscles of the shoulder; the *external respiratory,* to the outside of the chest."

To sum up.—We repeat that this is an excellent book; that it contains as much real doctrinal, as well as practical, information on human anatomy as we should wish every medical man's mind to be stored with; that it will certainly supersede all other books of this class, for it even contains copious directions for making preparations; that it does infinite credit to Mr. Shaw; and that it is not much to say, that a second edition will be called for as soon as the numerous pupils, who are thronging to the mart of medical knowledge at the opening of the winter season, shall have felt and duly appreciated its real value.

Whenever another edition shall be called for, we hope it will display a little more of the *lucidus ordo.* It cannot be a matter of difficulty for Mr. Shaw to give to the contents of his book the appearance of a better arrangement than there is at present.

